# Diagnostic Performance of Donor-Derived Plasma Cell-Free DNA Fraction for Antibody-Mediated Rejection in Post Renal Transplant Recipients: A Prospective Observational Study

**DOI:** 10.3389/fimmu.2020.00342

**Published:** 2020-02-28

**Authors:** Huanxi Zhang, Chunting Zheng, Xirui Li, Qian Fu, Jun Li, Qun Su, Liuhong Zeng, Zu Liu, Jiali Wang, Huiting Huang, Bowen Xu, Mingzhi Ye, Longshan Liu, Changxi Wang

**Affiliations:** ^1^Organ Transplant Center, The First Affiliated Hospital, Sun Yat-sen University, Guangzhou, China; ^2^BGI-Guangzhou Medical Laboratory, BGI-Shenzhen, Guangzhou, China; ^3^Department of Nephrology, The First Affiliated Hospital, Sun Yat-sen University, Guangzhou, China; ^4^BGI Genomics, BGI-Shenzhen, Shenzhen, China; ^5^Guangdong Provincial Key Laboratory on Organ Donation and Transplant Immunology, Guangzhou, China; ^6^Guangdong Provincial International Cooperation Base of Science and Technology (Organ Transplantation), Guangzhou, China

**Keywords:** kidney transplantation, antibody-mediated rejection, donor-specific antibodies, donor-derived cell-free DNA, area under the curve, sensitivity, specificity

## Abstract

**Objective:**

To evaluate the diagnostic performance of donor-derived plasma cell-free DNA (cfDNA) in discriminating antibody-mediated rejection (ABMR) or *de novo* donor-specific antibodies (DSA) without histological lesions in kidney allograft recipients.

**Methods:**

In this prospective single center observational study, we enrolled kidney allograft recipients between November, 2016 and September, 2017 at the First Affiliated Hospital of Sun Yat-sen University. Kidney allograft recipients with ABMR, *de novo* DSA but no histological lesions or negative DSA, and stable renal function were included. The plasma cfDNA fraction was measured using a targeted, single nucleotide polymorphism (SNP)-based assay. Pathological diagnosis was made according to the 2015 Banff Kidney Rejection Classification. The area under the ROC curve (AUC-ROC) was determined using the bootstrapping method to estimate median and 95% confidence interval (95% CI). The sensitivity, specificity and Youden index, positive predictive value (PPV), and negative predictive value (NPV) were calculated for specific cfDNA fractions.

**Results:**

Totally 37 consecutive patients received kidney allografts, including 18 recipients in the ABMR group and 19 recipients in the stable allograft group (7 DSA-positive and 12 DSA-negative). All patients in the ABMR group were DSA positive and 7 patients in the stable group were DSA positive but had no pathologically proven ABMR. The median donor-derived plasma cfDNA fraction was 2.4% (Q1 1.52% -Q3 3.70%) in the ABMR group, and was significantly higher than that of the stable group (0.65%, Q1 0.57% -Q3 0.97%; *P* < 0.001), but comparable with that of the DSA-positive patients in the stable allograft group (*P* = 0.074). The AUC-ROC of cfDNA was 0.90 (95% CI, 0.79–0.98). When a cfDNA threshold of 1% was chosen, it had a sensitivity of 88.9% and a specificity of 73.7%. The PPV was 76.2% and the NPV was 87.5%.

**Conclusion:**

Donor-derived plasma cfDNA fraction increased in kidney allograft recipients with ABMR. Detection of donor-derived plasma cfDNA fraction may contribute to the discrimination between ABMR and stable renal allograft function and may aid early recognition of earlier stage antibody-mediated injury.

## Introduction

Antibody-mediated rejection (ABMR) is an important determinant of long-term outcome of kidney allografts (1). Currently, kidney needle biopsy, though invasive and cost ineffective, remains the gold standard for diagnosis of ABMR. The procedure is clinically underused for surveillance of kidney allograft injury and is utilized for confirmation of suspected kidney allograft injury as indicated by rising serum creatinine levels which are converted to the estimated glomerular filtration rate (eGFR). However, serum creatinine often leads to delayed or missed diagnosis of ABMR due to its suboptimal sensitivity and specificity. Furthermore, subclinical ABMR cannot be detected by monitoring serum creatinine. Circulating donor specific antibodies (DSAs) against human leukocyte antigens (HLA) promote ABMR development (2). Nevertheless, only 30–40% DSA positive kidney allograft recipients develop ABMR (3). More sensitive and non-invasive markers of early ABMR are urgently needed in order to provide prompt diagnosis and tailored therapy of ABMR.

Previous studies have demonstrated the presence of plasma donor-derived cell-free DNA (cfDNA) in allograft recipients (4), which can be a candidate non-invasive marker of allograft injury or rejection (5–8). Measurement of donor cfDNA offers promise in non-invasive diagnosis of ABMR. Donor cfDNA is derived from DNA fragments released from necrotic or apoptotic cells in injured donor tissues (9). Donor cfDNAs possess identical properties to those of cfDNA in general: they are approximately 85–200 base pairs in size (10), and are metabolized and cleared in the liver (9, 11) independent of the renal function, which is probably due to their negative ions rendering them non-filterable through the glomeruli (12). Donor cfDNA levels rise in solid allografts following ischemia-reperfusion injury (8, 13, 14).

Only scant literature is available on donor-derived cfDNA in kidney allografts. Studies have shown that donor-derived cfDNA levels are similar in renal function stable kidney allograft recipients and heart transplant recipients (7, 15). A small single center study revealed that high cfDNA levels were associated with acute rejection (6). The role of donor-derived cfDNA in ABMR of kidney allografts is still ill defined. The onset and development of ABMR evolve from DSA generation to pathologic injury and then to outright clinical manifestations (16). In clinical practice, treatment is initiated at the time when pathologic injury, and DSAs are detected in transplant recipients. Furthermore, the efficacy of currently available and new therapeutic protocols is still inconclusive (17, 18). Donor-derived cfDNA can be a more timely injury marker than needle biopsy. In addition, few studies have investigated the relationship between donor-derived cfDNA and severity of pathologic injury and prognosis of ABMR.

We developed a single nucleotide polymorphism (SNP)-based method for detecting donor cfDNA, and have confirmed its use in monitoring acute rejection and infection in lung transplant patients (19). In the current prospective study, we sought to assess the diagnostic performance of donor-derived cfDNA for discriminating ABMR and stable graft function patients, and further evaluate the correlation between donor-derived cfDNA and pathological severity and prognosis of ABMR.

## Patients and Methods

### Patients

In this prospective single center observational study, we enrolled consecutive kidney allograft recipients who received care between November, 2016 and September, 2017 at the First Affiliated Hospital of Sun Yat-sen University, Guangzhou, China. The stable allograft group consisted of kidney allograft recipients (1) who had no proteinuria, within the preceding year or from discharge from the hospital to enrollment in the study, whichever was shorter, (2) whose eGFR was greater than 40 mL/min⋅1.73 m^2^ and fluctuated within ±20% of the mean eGFR within the preceding year or from discharge from the hospital to enrollment in the study, whichever was shorter, and (3) who were HLA antibody negative, or who were DSA positive but had normal histology or no apparent pathological changes on percutaneous kidney biopsy. The ABMR group consisted of kidney allograft recipients (1) who were DSA positive, (2) who were indicated for percutaneous kidney biopsy because of elevated creatinine, proteinuria, positive DSA or follow-up examination after treatment, and (3) who had pathologic changes consistent with ABMR. The eGFR was determined using MDRD (≥16 years) or Schwartz (<16 years).

The study protocol was approved by the local ethics committee at the authors’ affiliated hospital [No. (2017)171]. All study participants provided written informed consent. Kidney allografts from living or deceased organ donors who met the ethical guidelines for kidney donation were used.

### Blood Samples and cfDNA Measurement

Whole peripheral venous blood (5 mL) was collected at admission, 6, 12, and 24 months post kidney allograft surgery or at the time of each kidney allograft biopsy. Whole blood was centrifuged for 10 min at 1600 *g* 4°C within 4 hours of collection. The plasma supernatant was further clarified by centrifugation for 10 min at 16000 *g* to remove any remaining cells. The cells and the clarified plasma were stored at −80°C until use.

Plasma cfDNA was isolated using the QIAmp Circulating Nucleic Acid Kit (Qiagen, Hilden, Germany) according to the manufacturer’s protocol. We measured cfDNA using a targeted next-generation sequencing assay (19) that employs 56049 SNPs to accurately quantify cfDNA in transplant recipients without need for separate genotyping of the recipient or the donor. The cfDNA assay is precise across the linear quantifiable range (0.5–8% cfDNA) with a mean across-run coefficient of variation of 7.9%. The donor-derived cfDNA fraction was calculated as percentage cfDNA using a weighted formula (20). All measurements were performed by staff unaware of the identity of the samples.

### HLA Matching

Cellular DNA was extracted using DNeasy Blood & Tissue Kit (Qiagen) as instructed by the manufacturer. HLA alleles (HLA-A, -B, and -C, and class II HLA-DRB1, -DQA1, -DQB1, -DPA1, and -DPB1) were detected using the Luminex platform and sequence-specific oligonucleotide (SSO) technique using the LIFECODES HLA-SSO kit (Immucor Transplant Diagnostics, United States) as instructed by the manufacturer. Specific sequences were analyzed using MATCHIT!^TM^ DNA software (version 1.2, Immucor GTI Diagnostics) to determine HLA genotype.

### Detection of Anti-HLA Antibodies

Anti-HLA antibodies including antibodies against class IHLA-A, -B, and -C, and class II HLA-DRB1, -DQA1, -DQB1, -DPA1, and -DPB1 antigens were detected using the Luminex platform (Immucor Transplant Diagnostics) as instructed by the manufacturer. The mean fluorescence intensity of HLA antibodies was then calculated by normalization against the negative control. Data were analyzed using the LIFECODES MATCHIT!^TM^ ANTIBODY software(version 1.2, Immucor Transplant Diagnostics). A mean fluorescence intensity <1000 was considered negative, between 1000 and 4000 weakly positive, between 4000 and 10000 intermediately positive, and >10000 strongly positive.

### Pathological Diagnosis

Pathological diagnosis of rejection was made according to the 2015 Banff Kidney Rejection Classification (21) by two experienced pathologists (YS and CW) who were blind to the cfDNA results. C4d in transplant renal tissues was detected by immunofluorescence on frozen sections. Histological sections were categorized as (1) normal or unapparent lesion, (2) ABMR, (3) borderline changes, (4) T cell mediated rejection (TCMR), (5) interstitial fibrosis and renal tubule atrophy, and (6) other lesions unrelated to acute and chronic rejection according to the Banff Working Group (21).ABMRwas classified as acute active or chronic active ABMR. ABMR could be concurrent with TCMR, borderline changes, interstitial fibrosis and renal tubular atrophy, and other lesions unrelated to acute and chronic rejection.

### Treatments

Clinicians who were blinded to the cfDNA results chose treatment protocols for ABMR based on clinical conditions. Treatments included one or more of the following: plasma exchange, intravenous immunoglobulin, rituximab, and bortezomib. Glucocorticoid pulse therapy or anti-thymocyte globulin (ATG) was given if concurrent TCMR was present. The kidney allograft function-stable group and the DSA group were closely monitored and did not receive specific treatment. If ABMR developed, they were treated as described above.

### Patient Evaluation

In additional to renal biopsy, routine blood tests and urinary test, biochemistries and hepatic and renal function tests were done regularly. Measurement of plasma concentrations of immunosuppressive drugs and anti-HLA antibodies were undertaken regularly. Complications, loss of graft function and death were recorded.

### Statistical Analysis

All statistical analysis was done using R for Windows 3.6.1. Continuous variables were expressed as mean ± SD if normally distributed or median (the first quartile Q1 – the third quartile Q3) if non-normally distributed. Categorical data were expressed as frequency and percentage. Student’s *t* test was used for comparing differences between two groups for normally distributed data; Mann-Whitney U test was used for non-normally distributed data. One-way ANOVA or Kruskal-Wallis H test was used for comparison of continuous variables among more than two groups. A mixed linear model was used for repeated measurements of eGFR. *P* < 0.05 was considered statistically significant. Correlation between cfDNA and eGFR, and DSA MFI was evaluated by Spearman correlation analysis. The diagnostic performance of the cfDNA fraction for ABMR was evaluated using the receiver operating characteristic (ROC) curve. The area under the ROC curve (AUC-ROC) was determined using the bootstrapping method to estimate median and 95% confidence interval (95% CI). The sensitivity, specificity and Youden index, positive predictive value (PPV) and negative predictive value (NPV) were calculated for specific cfDNA fractions (1, 2, 3, 4, 5, 6, and 7%). The optimal threshold was the cfDNA fraction corresponding to the maximal Youden index:

Youden⁢index=sensitivity+specificity-1.

## Results

### Demographic and Baseline Characteristics of Kidney Allograft Recipients

**FIGURE 1:**
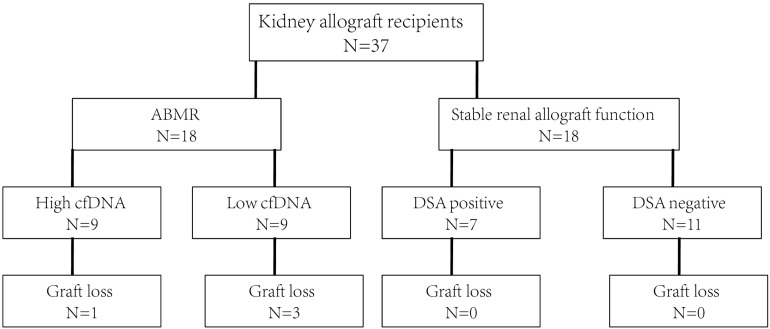
The study flowchart. ABMR, antibody mediated rejection; cfDNA, cell free DNA; DSA, donor specific antibody.

The study flowchart is shown in Figure 1. Totally 37 consecutive patients received kidney allografts during the study period. There were 19 recipients in the stable allograft group including 12 DSA negative recipients and 7 DSA positive recipients with no pathologically proven ABMR. There were 18 DSA positive recipients in the ABMR group. Their mean age was 35.8 ± 12.3 years and 70.3% of them were female. The demographic and baseline characteristics of kidney allograft recipients are shown in Table 1. The ABMR group had significantly longer median post-allograft duration (3.17 years, 2.33 – 7.39) than the stable allograft group (1.05 years, 0.48 – 4.03) (*P* = 0.045). The ABMR group had significantly higher mean fluorescence intensity of DSA than the stable allograft group (*P* < 0.001). Furthermore, the ABMR group had a significantly lower mean eGFR than the stable allograft group (50.5 ± 22.7 vs. 66.8 ± 14.1; Student’s *t* test, *P* = 0.014). The two groups were comparable in other demographic and baseline variables.

**TABLE 1:** Demographic and baseline characteristics of the kidney allograft recipients.

**Variables**	**ABMR**	**Stable allograft group**	***P***
*N*	18	19	
Mean (SD) age, years	39.4 (13.1)	32.5 (10.7)	0.090
Male gender, *n* (%)	6 (33.3)	5 (26.3)	0.915
Time post-allograft, years			**0.045**
Median (Q1, Q3)*	3.17(2.33,7.39)	1.05(0.48,4.03)	
Primary disease, *n* (%)			0.172
Diabetic nephropathy	1 (5.56)	0 (0.00)	
FSGS	0 (0.00)	1 (5.26)	
IgA nephropathy	3 (16.7)	1 (5.26)	
IgM nephritis	1 (5.56)	0 (0.00)	
Polycystic nephropathy	1 (5.56)	0 (0.00)	
Unknown	12 (66.7)	17 (89.5)	
Re-allograft	0	0	.
History of diabetes, *n* (%)			0.486
Yes	1 (5.56)	0 (0.00)	
History of transfusion, *n* (%)			0.660
Yes	3 (16.7)	2 (10.5)	
Pre-allograft DSA, *n* (%)			0.486
Positive	1 (5.56)	0 (0.00)	
Donor type, *n* (%)			0.879
Deceased	9 (50.0)	8 (42.1)	
Living	9 (50.0)	11 (57.9)	
HLA mismatch, *n* (%)			0.416
≥4	1 (5.56)	3 (15.8)	
<4	8 (44.4)	10 (52.6)	
Unknown	9 (50.0)	6 (31.6)	
eGFR, mean (SD)	50.5 (22.7)	66.8 (14.1)	**0.014**
Urinary protein, *n* (%)			0.125
0	12 (66.7)	18 (94.7)	
1+	3 (16.7)	1 (5.26)	
2+	1 (5.56)	0 (0.00)	
3+	2 (11.1)	0 (0.00)	
DSA MFI, *n* (%)			**<0.001**
Negative (<1000)	0 (0.00)	12 (63.2)	
Weak (1000–4000)	4 (22.2)	4 (21.1)	
Intermediate (4000–10000)	4 (22.2)	1 (5.26)	
Strong (>10000)	10 (55.6)	2 (10.5)	

DSA, donor-specific antibody; FSGS, focal segmental glomerulosclerosis; MFI, mean fluorescence intensity. *Q1, Q3 represent the first (25th percentile) and third (75th percentile) quartiles, respectively. A *P* value in bold type denotes a significant difference (*p* < 0.05).

In addition, the Banff pathologic lesions of the ABMR groups are shown in Table 2. Nine recipients were C4d negative (C4d 0 or C4d 1 in immunofluorescence staining), 11 (11/18, 61.1%), and 7 (7/18, 38.9%) recipients had acute active and chronic active ABMR, respectively. Six (6/18, 33.3%) recipients had IgA nephropathy, 1 (1/18, 5.56%) recipient had borderline changes and 1(1/18, 5.56%) recipient had calcineurin inhibitor toxicity.

**TABLE 2:** Banff lesion grades in the antibody mediate rejection group, *n* (%).

	**ABMR**		**ABMR**
*N*	18		18
**g**		**C4d**	

0	2 (11.1%)	0	5 (27.8%)
1	9 (50.0%)	1	4 (22.2%)
2	3 (16.7%)	2	4 (22.2%)
3	4 (22.2%)	3	5 (27.8%)

**ptc**		**cg**	

0	1 (5.56%)	0	11 (61.1%)
1	8 (44.4%)	1	4 (22.2%)
2	8 (44.4%)	2	1 (5.56%)
3	1 (5.56%)	3	2 (11.1%)

**i**		**ci**	

0	12 (66.7%)	0	4 (22.2%)
1	5 (27.8%)	1	8 (44.4%)
2	1 (5.56%)	2	6 (33.3%)
3	0	3	0

**t**		**ct**	

0	10 (55.6%)	0	5 (27.8%)
1	7 (38.9%)	1	7 (38.9%)
2	1 (5.56%)	2	6 (33.3%)
3	0	3	0

**v**		**cv**	

0	16 (88.9%)	0	8 (44.4%)
1	2 (11.1%)	1	7 (38.9%)
2	0	2	3 (16.7%)
3	0	3	0

*ABMR, antibody mediated rejection; cg, glomerular double contours; ci, Interstitial fibrosis; ct, tubular atrophy; cv, vascular fibrous intimal thickening; g, glomerulitis; i, inflammation; ptc, peritubular capillary; t, tubulitis; v, intimal arteritis.*

### Donor-Derived Plasma cfDNA Fractions by Groups

**FIGURE 2:**
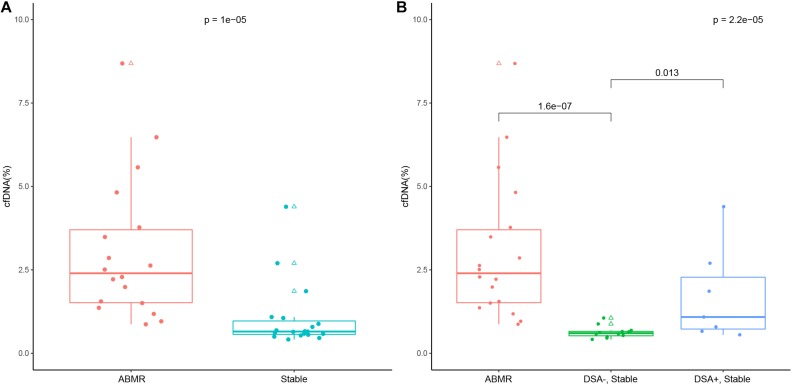
Donor-derived cell free DNA (cfDNA) fraction in the ABMR group and the stable allograft group **(A)**, and in the ABMR group, the DSA-positive and negative stable allograft subgroup **(B)**. The lower and upper whisker of the box whisker plot represent 5% and 95%, and the upper and lower border of the box represent 25th and 75th percentile and the mid transverse line represents the median. Solid dots represent true values and triangles represent outliers (>95th percentile and <5th percentile).

The median donor-derived plasma cfDNA fraction was 2.4% (1.52–3.70%) in the ABMR group, and was significantly higher than that of the stable allograft group (0.65%, 0.57–0.97%; *P* < 0.001, Figure 2A). Meanwhile, the median donor-derived plasma cfDNA fraction in DSA-positive patients in the stable allograft group was significantly higher than that of DSA-negative patients of the same group (1.09%, 0.73–2.28% vs. 0.60%, 0.53–0.66%; *P* = 0.016) (Figure 2B). However, the median donor-derived plasma cfDNA fraction in the ABMR group was comparable with that of the DSA-positive patients in the stable allograft group (*P* = 0.074).

### The Relationship Between cfDNA Level and Prognosis

**FIGURE 3:**
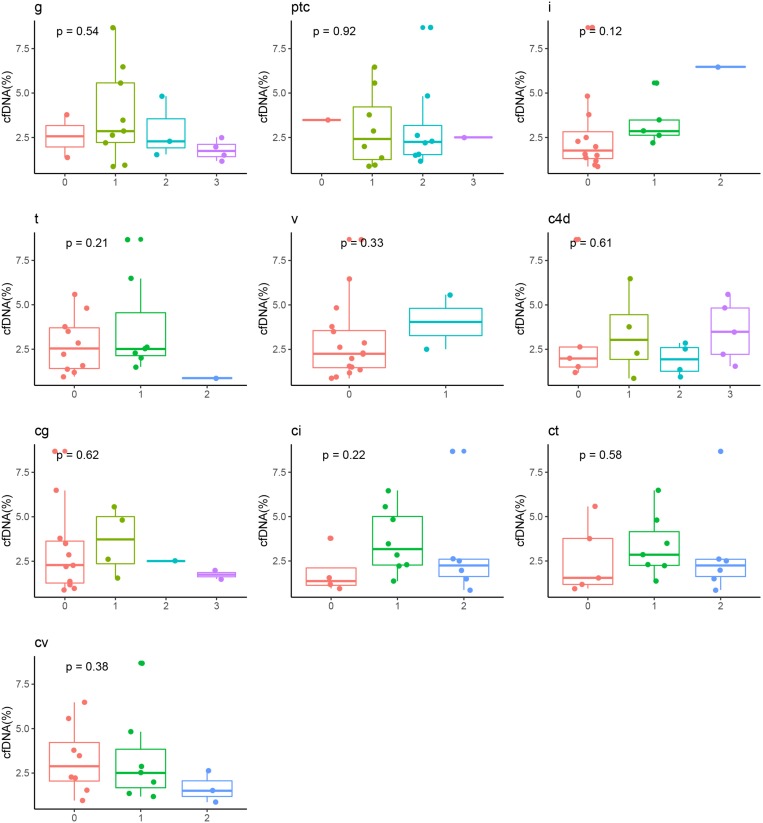
Correlation between cfDNA fraction and grades of Banff pathologic categories in ABMR recipients. ABMR, antibody mediated rejection; cg, glomerular double contours; ci: Interstitial fibrosis; ct, tubular atrophy; cv, vascular fibrous intimal thickening; g, glomerulitis; i, inflammation; ptc, peritubular capillary; t, tubulitis; v, intimal arteritis.

The median time from renal transplant surgery to study enrollment was 3 years (range 1.0 – 4.4 years). The patients were followed up for median duration of 1.6 years (range 1.5 – 1.8 years). No patients in the cohort were lost to follow up. Except at the baseline, the eGFR was comparable among the ABMR group, DSA (+) subgroup and DSA (−) subgroup in the stable allograft group (*P* > 0.05) (Table 3). Four (4/18, 22.2%) patients in the ABMR group had graft loss while no patient in the stable allograft group had graft loss. In addition, the cfDNA fraction exhibited no statistically significant correlation with the mean fluorescence intensity of DSA in the ABMR group (*r* = 0.15, *P* = 0.54). Furthermore, there was no statistical difference in cfDNA fractions among patients in the ABMR group with different Banff lesion grades (Figure 3). There was also no statistically significant difference in cfDNA fractions between acute and chronic ABMR patients (2.29%, 1.18–3.77% vs. 2.51%, 1.55–4.82%, *p* = 0.791).

**TABLE 3:** Kidney transplant outcome of the study cohort.

	**ALL**	**ABMR**	**Stable allograft group**		***P***
			**DSA+**	**DSA−**	
*N*	37	18	7	12	
eGFR					
Baseline	58.8 (20.3)	50.5 (22.7)	72.4 (21.9)	63.5 (5.59)	**0.028**
6 months^#^	60.4 (25.6)	54.6 (31.6)	73.3 (25.1)	61.4 (9.71)	0.262
1 year^#^	58.8 (23.9)	49.3 (28.2)	68.5 (20.9)	67.3 (10.9)	0.059
2 years^#^	59.7 (29.7)	52.2 (38.9)	73.2 (21.6)	63.6 (10.6)	0.290
Graft loss, *n* (%)					0.115
Yes	4 (10.8)	4 (22.2)	0 (0.00)	0 (0.00)	

*eGFR, estimated glomerular filtration rate. ^#^Time from cfDNA detection. A *P* value in bold type denotes a significant difference (*p* < 0.05).*

**FIGURE 4:**
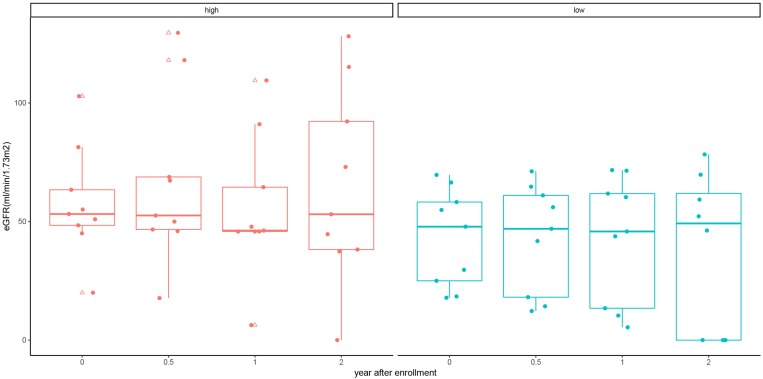
eGFR of kidney allograft recipients with antibody mediated rejection at 6, 12, and 24 months stratified by high and low cfDNA fraction. Solid dots represent true values and triangles represent outliers (> 95th percentile and > 5th percentile).

We further used the median cfDNA fraction (2.4%) to categorize ABMR recipients into the low and high cfDNA fraction subgroup. Though the eGFR was numerically higher in the high cfDNA fraction subgroup than the low cfDNA fraction subgroup at each follow up measurement, no statistical difference was observed (*P* > 0.05) (Table 4 and Figure 4). Furthermore, 3 patients in the low cfDNA fraction subgroup and 1 patient in the high cfDNA fraction subgroup had graft loss. The pathological score of the four recipients that lost their grafts was shown in Table 5.

**TABLE 4:** Kidney transplant outcome in the ABMR group stratified by cfDNA fractions.

	**cfDNA fractions**		***P***
	**High**	**Low**	
N	9	9	
Median (Q1, Q3) cfDNA (%)*	3.77 (2.86;5.57)	1.50 (1.18;1.98)]	**<0.001**
eGFR			
Baseline	57.8 (23.4)	43.1 (20.6)	0.177
6 months^#^	66.3 (35.9)	42.9 (22.8)	0.122
1 year^#^	55.9 (29.8)	42.7 (26.6)	0.336
2 years^#^	64.7 (41.2)	38.2 (33.1)	0.164
Graft loss			0.576
Yes	1 (11.1%)	3 (33.3%)	

eGFR, estimated glomerular filtration rate. ^#^Time from cfDNA detection. *Q1, Q3 represent the first (25th percentile) and third (75th percentile) quartiles, respectively. A *P* value in bold type denotes a significant difference (*p* < 0.05).

**TABLE 5:** The pathological score and donor-derived cfDNA fraction of the four recipients that lost their graft.

**ID**	**cfDNA%**	**g**	**ptc**	**i**	**t**	**cg**	**ci**	**ct**	**v**	**cv**	**c4d**
1	0.87	1	1	0	2	0	2	2	0	2	1
2	1.50	3	2	0	1	3	2	2	0	2	0
3	1.98	3	1	0	1	3	2	2	0	1	0
4	5.57	1	1	1	0	1	1	0	1	0	3

### Diagnostic Performance of cfDNA Fraction for ABMR

**FIGURE 5:**
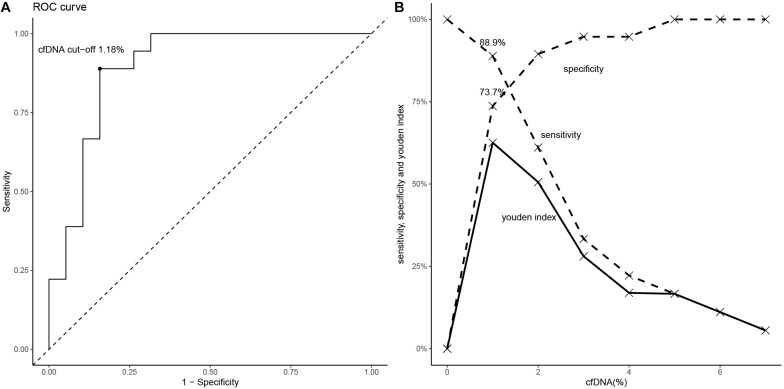
The diagnostic performance of cfDNA fraction for ABMR. **(A)** The ROC (if the cfDNA threshold is 1.18%, the Youden index is maximal). **(B)** The values of sensitivity, specificity and Youden index are shown according to specific cfDNA thresholds.

To define the AUC-ROC performance of cfDNA, we included all cfDNA results of the 37 allograft recipients. The AUC-ROC of cfDNA was 0.90 (95% CI, 0.79–0.98, boot = 1000) (Figure 5A). When a cfDNA threshold of 1% was chosen, it had a sensitivity of 88.9% and a specificity of 73.7%. The PPV was 76.2% and the NPV was 87.5% (Figure 5B). The ROC-AUC for discriminating ABMR from stable allograft function was 0.74 (95% CI, 0.48 to 0.99) in DSA + patients.

## Discussion

Our prospective observational study revealed that donor-derived plasma cfDNA fraction well discriminated ABMR from stable renal allograft function in post kidney allograft transplant recipients. The ABMR group had a significantly higher median cfDNA fraction than the stable allograft group. The DSA negative patients in the stable allograft group had the lowest median cfDNA fraction. Our ROC analysis further showed an AUC of 0.90 (95% CI, 0.79–0.98), suggesting that donor-derived plasma cfDNA fraction is of good diagnostic performance.

At the cfDNA fraction optimal threshold of 1% for ABMR, the test had a sensitivity of 88.9% and a specificity of 73.7%, a PPV of 76.2%,and a NPV of 87.5%, which is similar to the findings by Bloom et al. who identified an AUC-ROC value of 0.74 (95% CI, 0.61–0.86) for donor-derived cfDNA fraction in discriminating active rejection including TCMR and ABMR and no active rejection and a AUC-ROC value of 0.87 (95% CI, 0.75–0.97) in discriminating between ABMR and no ABMR and the optimal threshold for discriminating ABMR is 1.0%, with a PPV of 44% and a NPV of 96% (22). Oellerich et al. studied 189 kidney allograft recipients and found that donor-derived cfDNA fraction had an AUC-ROC value of 0.73 in discriminating between recipients with rejection and those with stable renal allograft function: at a threshold of 0.43%, the sensitivity was 73%, and the specificity was 69%, the PPV was 12%, and the NPV was 98% (23). These investigators showed that the absolute donor-derived cfDNA value was superior to cfDNA fraction in discriminating rejection and stable allograft function, with an AUC-ROC value of 0.83. At a threshold 52 cp/mL, it had a sensitivity of 73%, a specificity of 73%, a PPV of 13%, and an NPV of 98%. They believed that cfDNA fraction was subject to influences by recipient cfDNA levels which tended to increase during infection, exercise and medications, thus reducing donor-derived cfDNA fraction. In addition, most plasma cfDNA is derived from apoptotic leukocytes such as neutrophils and lymphocytes. Donor-derived cfDNA absolute value can avoid these influences. Sigdel et al. (24) carried out kidney needle biopsy of 217 patients and found that cfDNA fraction had an AUC-ROC value of 0.87 (95% CI, 0.80–0.95) in discriminating between active rejection and non-rejection. At a threshold of 1%, it had a sensitivity of 88.7%, a specificity of 72.6%, with a PPV of 52% and an NPV of 95%. The median cfDNA fraction was 2.3% and 0.4% for rejection and stable renal allograft function patients, respectively. Huang et al. (25) assessed 63 adult kidney transplant recipients with suspicion of rejection with donor-derived cfDNA and allograft biopsy and found that the AUC-ROC value for active rejection was 0.71 (95% CI, 0.58–0.85). For ABMR, the AUC was 0.82 (95% CI, 0.71–0.93) and a donor-derived cfDNA fraction ≥ 0.74% yielded a sensitivity of 100%, specificity 71.8%, PPV 68.6%, and NPV 100%. E. Dauber at al. (26) measured ddcfNA in plasma samples from 29 kidney transplant recipients obtained at time of clinically indicated biopsies and revealed an AUC-ROC for discriminating acute rejection and non-acute-rejection biopsies of 0.84 (95% CI: 0.66–1.00). The determined cutoff value of 2.7% donor-derived cfDNA showed a sensitivity of 0.88 (95% CI, 0.63–1.00) and specificity of 0.81 (95% CI, 0.64–0.98). However, Gielis et al. (27) presented the contradictory results and they found that plasma ddcfDNA did not outperform the diagnostic capacity of the serum creatinine in the diagnosis of acute rejection.

Our study and three other studies all showed that cfDNA fraction has a high NPV for ABMR diagnosis, which means most of patients with negative cfDNA fraction (below the threshold) do not have ABMR, suggesting that donor-derived cfDNA fraction measurement may avoid unnecessary kidney transplant needle biopsy when the other types of allograft injury can be excluded, especially in patients on anticoagulation medication or patients for whom it is inconvenient to perform kidney needle biopsy. For example, for patients with positive DSA but no clinical manifestations, if cfDNA fraction is negative, biopsy may be avoided temporarily and close follow-up should be performed.

It remains controversial whether ABMR and TCMR could be discriminated by cfDNA fraction. Bloom et al. (22) found that cfDNA fraction was higher in ABMR group compared with TCMR group and cfDNA fraction tended to be higher in TCMR types ≥ IB than type IA, suggesting the association of cfDNA fraction with the severity of TCMR. Huang et al. (25) supported this conclusion and demonstrated that cfDNA fraction was higher in ABMR compared with TCMR while cfDNA fraction was not able to discriminate TCMR from no rejection. However, Oellerich et al. (23) and Sigdel et al. (24) showed that cfDNA fraction increased in either ABMR or TCMR and it was unable to discriminate them. More studies with large sample size were needed to solve the problem.

Up to now, the diagnostic performance of cfDNA as a complement to DSA detection and biopsy in ABMR is not clear. Jordan et al. carried out a secondary analysis of the DART study (28) that included 87 kidney allograft recipients whose DSA was monitored (ABMR: 16 cases). They found that the median donor-derived cfDNA fraction was 2.9% for DSA positive kidney allograft recipients who developed ABMR versus 0.34% for those who did not, which is similar to that of DSA negative patients (0.29%). In DSA positive patients, the AUC-ROC of cfDNA for discriminating ABMR was 0.86. When the threshold was 1%, the cfDNA fraction had a PPV of 81% and an NPV of 83%, while simple DSA positivity had a PPV of 48% for ABMR. They concluded that combination of donor-derived cfDNA fraction and DSA could improve non-invasive diagnosis of ABMR. In DSA positive patients, cfDNA fraction above the threshold could be used for diagnosing ABMR, which had better performance compared with in all the kidney allograft recipients with indicational biopsy (PPV 44%) (22). In 2019, Huang et al. (25) also conducted a study with similar purpose, in which there were 7 patients with histologic findings suspicious for ABMR on biopsy who did not meet Banff 2013 criteria for ABMR given the absence of DSA. Only 2 of these 7 patients had a cfDNA <1.0%; the remaining 5 had high levels of cfDNA that ranged from 1.3 to 7.7%. It is tempting to consider that the cfDNA test may potentially identify cases of histologic ABMR without detectable DSA. Here, ABMR is likely mediated by non-HLA DSAs such as antibodies to the type 1 angiotensin receptor (anti-AT1R). The design of our study is different from the above two studies. In the above two studies, DSA positive patients without ABMR had the other types of pathological lesions as well as the clinical manifestations in various extent. It was difficult to infer what type of lesions caused the elevation of cfDNA in the DSA + ABMR- group. However, in our study, DSA positive patients without ABMR were confirmed to have neither pathological damage nor clinical manifestations and other complications such as vascular complications were excluded. Our result showed that 4 in 7 DSA positive patients without any evidence of pathological lesions had a donor-derived cfDNA fraction >1%, which made the distribution of cfDNA in this group similar to the distribution among those with ABMR. One likely explanation is that kidney needle biopsy may have failed to detect occult DSA mediated kidney injury, suggesting that donor-derived cfDNA fraction may recognize earlier stage antibody mediated injury, thus providing evidence for early initiation of therapy. However, it should also be noted that there were only 7 cases in DSA-positive subgroup of the stable allograft group, and the median dd-cfDNA level of this subgroup was lower than that of ABMR group without statistical difference. It is quite possible that increasing the number of cases may result in significant difference. Interestingly, we found that there was large variation in the donor-derived cfDNA fraction in this group. Therefore, the median value of cfDNA in DSA-positive subgroup of the stable allograft group may largely depend on the number of patients with biopsy undetectable DSA-induced injury. This needs confirmation by studies with a larger sample population.

Few studies have investigated the relationship between donor-derived cfDNA and severity of pathologic injury and prognosis of ABMR. Our study did not show any correlation between cfDNA fraction and the mean fluorescence intensity of DSA in ABMR recipients, Banff pathologic categories and prognosis of kidney transplant, indicating that cfDNA fraction may not be used for assessing severity of acute and chronic lesions in ABMR as well as prognosis of ABMR. There were 3 patients in the low cfDNA fraction subgroup and 1 patient in the high cfDNA fraction subgroup who had graft loss. Among the three patients with low cfDNA (< median 2.4%), the pathological damage was serious (Table 5), and thus their prognosis was poor. It needs more data to confirm this conclusion, and its mechanism has yet to be clarified.

Under the current evidence, dd-cfDNA cannot be used alone as a diagnostic tool for a certain disease, and the diagnosis needs to be made in combination with medical history, clinical manifestations and other test results. To better apply the published evidence to the clinical practice, there are several aspects requiring clarification. First, the study population will affect the results interpretation. The risk of ABMR is variable in different study population, which will have an impact on the performing characteristics of cfDNA in diagnosis of ABMR. Second, the accuracy of the pathological diagnosis may have an effect on the results. The biopsy, which remains the gold standard for diagnosis of ABMR, reflects just a small part of the lesions and the pathological diagnosis is closely related to the location of the puncture, the quality of the specimen, and the experience of the pathologist. Third, up to now, no studies have investigated the association of cfDNA and the type of DSA (HLA-DSA, non-HLA-DSA, IgG subtypes, complement-binding DSA, etc.). It has been realized that there is a difference in the pathogenicity of DSA, and 60–70% of patients with DSA do not develop ABMR. What causes the difference is still controversial. This is one of the directions for future research. However, it may be restricted by the high variability of DSA which requires large sample size and high cost to identify the effects. Fourth, detection timing will also affect the results. Evidence has confirmed that cfDNA will increase early in the postoperative period, and about 5 days after the will operation, cfDNA decrease to the normal value (23). If the ischemia-reperfusion injury is serious, the cfDNA will decline more slowly. Therefore, cfDNA needs to be used during the postoperative stable period. All the cases we included were in the stable phase, excluding the effect of acute kidney injury on the outcome.

Our study has several limitations. First, the sample size in this single-center study was not large enough. Nevertheless, the number of patients in the experimental groups (e.g., ABMR group, DSA + subgroup of the stable graft function group) was comparable with that of the previously published studies (25, 28). Second, to further define the discriminative power of cfDNA fraction for complications of kidney transplants, investigators should include more samples with the other pathological lesions in future studies.

## Conclusion

In conclusion, donor-derived plasma cfDNA fraction increased in kidney allograft recipients with ABMR and the detection of donor-derived plasma cfDNA fraction may contribute to the discrimination between the recipients with ABMR and those with stable renal allograft function. The optimal threshold of cfDNA fraction for diagnosing ABMR is 1%, with a PPV of 76.2% and an NPV of 87.5%. Donor-derived cfDNA fraction may aid early recognition of earlier stage antibody mediated injury.

## Data Availability Statement

The data that support the findings of this study have been deposited in the CNSA (https://db.cngb.org/cnsa/) of CNGBdb with accession code CNP0000807.

## Ethics Statement

The studies involving human participants were reviewed and approved by the Ethics Committee of the First Affiliated Hospital of Sun Yat-sen University. Written informed consent to participate in this study was provided by the participants and/or their legal guardian/next of kin.

## Author Contributions

HZ, MY, and LL designed the study. HZ, CZ, and XL analyzed data and co-wrote the manuscript. CZ, XL, QS, and HH performed the experiments. JL and QF enrolled patients and collected data. LZ and ZL performed bioinformatic analyses. JW and BX collected and cleaned data. MY, LL, and CW supervised the research and critically reviewed the manuscript. All authors revised and approved the final manuscript. MY and LL was responsible for the decision to submit for publication.

## Conflict of Interest

CZ, LZ, ZL, and MY were employed by the company BGI-Shenzhen. The remaining authors declare that the research was conducted in the absence of any commercial or financial relationships that could be construed as a potential conflict of interest.
